# Burden of primary sclerosing cholangitis in Sweden (2002–2020): Incidence, outcomes, healthcare utilization, and costs

**DOI:** 10.1097/HC9.0000000000000858

**Published:** 2025-12-12

**Authors:** Annika Bergquist, Nandita Kachru, Martina Aldvén, Oskar Ström, Helena Skröder, Emilie Toresson Grip, Hannes Hagström

**Affiliations:** 1 Department of Medicine, Huddinge, Karolinska Institutet, Stockholm, Sweden; 2 Division of Hepatology, Department of Upper GI Diseases, Karolinska University Hospital, Stockholm, Sweden; 3 Gilead Sciences, Inc., Foster City, California, USA; 4 Quantify Research AB, Stockholm, Sweden

**Keywords:** cholangitis, cohort studies, cost of illness, healthcare costs, healthcare resource use, liver diseases, retrospective studies

## Abstract

**Background:**

There is limited real-world evidence on the economic burden associated with primary sclerosing cholangitis (PSC). This study evaluated the prevalence, incidence, baseline characteristics, long-term outcomes, healthcare resource use (HRU), and healthcare costs associated with PSC in Sweden.

**Methods:**

Adults with PSC were identified in the Swedish National Patient Register from 2002 to 2020 using International Classification of Diseases, 10th revision codes for PSC (K83.0A) and/or cholangitis (K83.0) + inflammatory bowel disease (IBD) (K50/K51). The index date was defined as the date of the first (incident) PSC diagnosis. Patients were required to have a look-back period of ≥360 days (baseline) and a follow-up period of ≥30 days. Annualized mean HRU and healthcare costs (in 2021 euros) were calculated at baseline and during follow-up.

**Results:**

Overall, 4213 incident patients with PSC were included (mean age 48.4 y; 56.8% male; 73.0% with IBD). At baseline, few patients had cirrhosis (4.2%), hepatobiliary or pancreatic cancers (4.6%), or had undergone liver transplantation (1.0%). Median duration of follow-up was 5.7 years. Outpatient visits, number of hospitalizations and filled prescriptions, and length of inpatient stay significantly increased from baseline to end of follow-up, with a 117% increase in annualized mean total healthcare costs from €9442 to €20,487 (*p*<0.0001), with hospitalization being a primary driver. In total, 935 patients (22.2%) died. The 10-year risk of any complication (any malignancy, cirrhosis, or liver transplantation) was 25.9% (95% CI 24.0–27.9).

**Conclusions:**

HRU and healthcare costs for patients with PSC in Sweden were substantial and significantly increased after diagnosis. Effective therapies are needed to reduce disease progression and economic burden.

## INTRODUCTION

Primary sclerosing cholangitis (PSC) is a rare, chronic liver disease characterized by biliary inflammation and fibrosis of both intrahepatic and extrahepatic bile ducts, which frequently progresses to cirrhosis, and is associated with an increased risk of developing biliary tract cancer (cholangiocarcinoma). Although the exact causes of PSC are unclear, genetic and environmental factors, as well as immune-mediated mechanisms, are thought to contribute to its etiopathogenesis.^1^^,^^2^

PSC is a rare disease, with an estimated prevalence of 0.22–16.2 per 100,000 persons and an incidence of up to 1.3 per 100,000 persons/year in the United Kingdom, North America, and Europe, according to a systematic literature review conducted in 2011.^3^ In Sweden, the adult prevalence of PSC was 16.2 per 100,000 persons in 2005, with an incidence of 1.2 per 100,000 persons/year.^4^

The clinical presentation of PSC varies widely from being asymptomatic to presenting with abnormal liver biochemistries, liver fibrosis/cirrhosis, or liver failure.^2^^,^^5^ PSC is strongly associated with autoimmune diseases, in particular inflammatory bowel disease (IBD), with ~70%–80% of patients having concurrent IBD.^2^^,^^6^^–^^8^ PSC also increases the risks of developing hepatobiliary, colorectal, pancreatic, and gallbladder cancers.^9^^–^^11^ The estimated annual incidence of cholangiocarcinoma is 0.2%–2.7% and is the most common cause of death in patients with PSC.^12^^–^^14^

The management of PSC primarily involves symptom control and monitoring for complications.^1^^,^^2^^,^^15^ Management of symptoms includes the use of medications such as ursodeoxycholic acid and immunosuppressants, although there is little evidence supporting their impact on disease prognosis.^12^^,^^16^^–^^18^ No pharmacological treatments have been proven to effectively slow the progression of PSC.^19^ Liver transplantation remains the only curative treatment for PSC, but disease recurrence has been reported in ~20% of patients and is associated with significant morbidity and mortality.^20^ Furthermore, half of the patients with disease recurrence will require retransplantation.^12^

PSC imposes a substantial healthcare burden owing to its chronic, progressive nature, high mortality and morbidity, and frequent need for liver transplantation.^9^^,^^21^ Given the absence of curative treatments, managing PSC involves symptom management and slowing disease progression, which can result in significant healthcare resource use (HRU) and costs. There is limited published evidence using real-world data on the characteristics, HRU, and healthcare costs in patients with PSC in Sweden. Understanding the economic burden of PSC is essential for informing healthcare policy and resource allocation. This study aimed to evaluate the incidence, prevalence, baseline characteristics, long-term outcomes, HRU, and healthcare costs associated with PSC in Sweden.

## METHODS

### Study design and data sources

**FIGURE 1:**
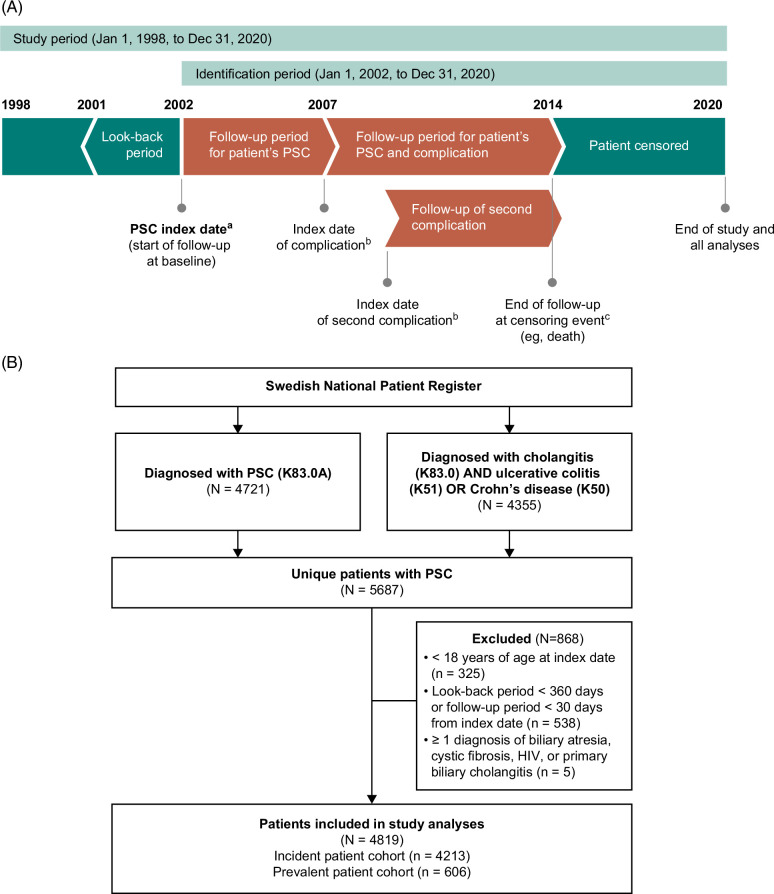
Study design and patient identification. (A) Hypothetical patient example in which PSC was diagnosed in January 2002, with a look-back period from January 2001. The patient was diagnosed with cirrhosis in January 2007 and later with colorectal cancer. Follow-up of PSC and complications continued until the patient's death in December 2014. (B) A flowchart of the identification of patients with PSC in Sweden between January 1, 2002, and December 31, 2020. ^a^Defined as the date of the first of at least 2 PSC diagnoses during the identification period. ^b^Defined as the diagnosis date of the complication(s) or death. Patients can be included in the follow-up of multiple complications. ^c^All patients were followed from the PSC index date until the date of the latest available data; censoring of patients occurred at death, emigration, or the end of the study period. Abbreviation: PSC, primary sclerosing cholangitis.

A nationwide, longitudinal cohort study was conducted using historical data from the Swedish National Patient Register (January 1, 1998 to December 31, 2020), the Cause of Death Register (January 1, 1998 to December 31, 2020), and the Prescribed Drug Register (July 1, 2005 to December 31, 2020) (Figure 1A). All 3 registers are maintained by the Swedish National Board of Health and Welfare.

The National Patient Register provides clinical, administrative, and demographic data for hospital inpatient care and outpatient specialist care. It includes primary and secondary diagnosis codes according to the International Classification of Diseases, 10th revision (ICD-10), as well as procedural codes and diagnosis-related group (DRG) codes, which were used to calculate costs related to each visit. The Cause of Death Register contains information on the date and underlying cause of death for all deceased individuals in Sweden and was used to extract data on all-cause death. The Prescribed Drug Register contains information on all dispensed prescriptions in Sweden, including the drug and the price of the drug, and was used to extract data on costs of filled prescriptions. Note that data on filled prescriptions were only available from July 1, 2005; therefore, previous medications could only be assessed at baseline for patients included after that date.

Ethical approval was obtained from the regional Ethical Review Board in Uppsala, Sweden (reference number: 2021-04422) before data extraction. The source data contained no direct identifiable patient information, and no informed consent was required for this study. The study was conducted in accordance with the Declaration of Helsinki and the International Society for Pharmacoeconomics and Outcomes Research (ISPOR) guidelines for good research practices in retrospective database analysis.

### Study population

Adult patients (≥18 y of age) with a primary or secondary recorded diagnosis of PSC from January 1, 2002, to December 31, 2020 were identified in the National Patient Register (identification period) using either the PSC-specific ICD-10 code K83.0A or a combination of the ICD-10 codes for cholangitis (K83.0) and IBD [Crohn disease (K50) or ulcerative colitis (K51)] (Figure 1B). The combination of the codes for cholangitis and IBD was used in addition to the PSC-specific code, because the implementation of the K83.0A code among physicians in Sweden over time was unknown and presumed to be low at the beginning of the study period (January 1998–December 2020). At least 2 separate visits for PSC and/or cholangitis and IBD diagnoses were required. These inclusion criteria are consistent with those used in a previous study of PSC conducted in Sweden.^22^ The index date was defined as the date of the first of these diagnoses during the identification period. Identified patients were required to have a look-back period of at least 360 days before the index date to allow for estimation of baseline variables and have a follow-up period of at least 30 days after the index date to exclude patients for whom detection of PSC coincided with terminal care.

Incident patients were defined as those with newly diagnosed PSC during the identification period (ie, without a PSC diagnosis during the look-back period) and were followed up from the index date to death, emigration, or the end of the study. Patients with a previous diagnosis of biliary atresia, cystic fibrosis, HIV, or primary biliary cholangitis were excluded.

### Study outcomes

Patient demographics and clinical characteristics were captured during baseline, covering all data up to and including the index date. Overall comorbidity burden at baseline was measured using the Quan–Charlson Comorbidity Index, based on all available data up to and including the index date, with higher scores indicating a greater burden of comorbidities and worse prognosis.^23^ Baseline complications, procedures, PSC-related comorbidities and medications, as well as all-cause HRU and healthcare costs, were evaluated in a predefined 360-day look-back period before index. Complications, HRU, and healthcare costs were also estimated during the follow-up period. Annualized HRU and costs were calculated by dividing total accumulated costs from the index date until censoring [emigration, death, or administrative study end (December 31, 2020)] by total follow-up time (years). This approach was chosen explicitly to capture multiple occurrences or recurrences of complications and costs along the disease trajectory. To further ensure robustness of results against censoring, we conducted sensitivity analyses examining the cumulative incidence of PSC-associated complications over time. HRU was measured as the annualized number of outpatient visits, hospitalizations, hospitalization days, and filled medication prescriptions in incident patients. Total healthcare costs were defined as the sum of annualized costs associated with outpatient visits, hospitalizations, and filled prescriptions, and were calculated based on DRG codes,^24^ care weights (standardized resource use for specific diagnostic combinations), and cost per unit of weight.

Costs of outpatient visits and hospitalizations were inflated to year 2021 costs by calculating an index of the average cost per patient over calendar years in the study period. Costs of filled prescriptions were adjusted for inflation by using the consumer price index for healthcare from Statistics Sweden. All costs were thus adjusted for inflation to 2021 values in Swedish kronor (SEK) and translated to euros (€) using the average annual exchange rates for 2021, as reported by the European Central Bank (EUR 1=SEK 10.1465).^25^ Costs in United States dollars (USDs) were additionally converted from SEK using the corresponding annual average exchange rate from the Swedish Central Bank (USD 1=SEK 8.58151)^26^ and are provided in Supplemental Table S1, http://links.lww.com/HC9/C196, for reference. Terminal care costs were calculated in the 360 days before death, which included outpatient visits, hospitalizations, and filled prescriptions.

Risks of complications of PSC and all-cause death among at-risk incident patients (those without the specified outcome at baseline) were calculated as the cumulative incidence of the first event (thus not allowing multiple events). Complications of PSC were defined as cirrhosis (compensated and/or decompensated), malignancy (cholangiocarcinoma, colorectal cancer, gallbladder cancer, HCC, pancreatic cancer), or liver transplantation.

### Statistical analysis

The incident patient cohort was used to assess patient characteristics, HRU, healthcare costs, and disease outcomes. Descriptive statistics were calculated for categorical variables (frequency and percentage) and continuous variables (mean, median, SD, and range). The paired Student *t* test was used for the comparison of continuous variables, where applicable. A 2-tailed *p*<0.05 was considered statistically significant. Descriptive analyses were performed for demographics and clinical characteristics. Annualized (total data divided by the number of years of follow-up) HRU at baseline and follow-up, and annualized healthcare costs at baseline and follow-up were analyzed. HRU included the number of outpatient visits, the number of hospitalizations, the days of inpatient stay, and the number of filled prescriptions. Healthcare costs were categorized under hospitalizations, outpatient visits, and filled prescriptions. The terminal cost of care was calculated over the 360 days before death. Observations without a DRG code were imputed as the mean cost for that period. A survival analysis was performed for the cumulative incidence of complications of PSC and all-cause death. Censoring of patients only occurred at death, emigration, or the end of the study period to allow for the follow-up of multiple complications. Stata 16 software (StataCorp LLC, College Station, TX, USA) was used for statistical analysis.

## RESULTS

### Study cohort characteristics

Between January 1, 2002, and December 31, 2020, 5687 unique patients with PSC were identified (Figure 1B). Of these, 868 patients did not meet the eligibility criteria and were excluded from the analyses. Overall, 4819 patients were included in the study cohort, which consisted of 4213 incident patients (those with no evidence of a PSC diagnosis before the index date) and 606 prevalent patients (those diagnosed with PSC before the start of the study period).

### Prevalence and incidence of PSC

In 2002, the crude prevalence of patients with PSC in Sweden was 10.5 per 100,000 persons (based on an adult population of 6,999,878). This had increased 4.2-fold by 2020 to a prevalence of 43.9 per 100,000 persons (based on an adult population of 8,189,892). As expected, PSC was more prevalent in men (14.6 per 100,000 men in 2002 and 51.0 per 100,000 men in 2020) than in women (6.6 per 100,000 women in 2002 and 36.9 per 100,000 women in 2020). The crude incidence of patients with PSC in Sweden increased 1.5-fold between 2002 and 2020, from 2.2 per 100,000 persons/year (2.9 for men and 1.6 for women) to 3.3 per 100,000 persons/year (3.3 for men and 3.3 for women).

### Baseline demographics and clinical characteristics

The mean age at diagnosis of incident patients was 48.4 years, and the majority were men (56.8%) (Table 1). Most patients (65.8%) had a Quan–Charlson Comorbidity Index score of 0–2. IBD was the most common comorbidity (73.0%). Approximately 10% of patients had a history of PSC complications, the most common of which was compensated cirrhosis (4.2%), followed by cholangiocarcinoma (1.5%), colorectal cancer (1.2%), and liver transplantation (1.0%). The most common liver-related procedure that patients had undergone at baseline was endoscopic retrograde cholangiopancreatography (7.4%). 5-aminosalicylic acid and sulfasalazine (30.0%) was the most common medication at baseline, followed by prednisolone (22.1%), ursodeoxycholic acid (16.4%), and azathioprine (10.1%).

**TABLE 1:** Baseline demographics and clinical characteristics at index date

	Incident patients (n=4213)
Sex, n (%)	
Male	2392 (56.8)
Age, y, mean (SD)	48.4 (18.86)
Age group, y, n (%)	
18–44	1966 (46.7)
45–64	1214 (28.8)
≥65	1033 (24.5)
Quan–Charlson Comorbidity Index, mean (SD)	2.3 (1.94)
Quan–Charlson Comorbidity Index, n (%)	
0	925 (22.0)
1–2	1849 (43.9)
3–4	1024 (24.3)
≥5	415 (9.9)
Patients with complications, n (%)	
No complications	3825 (90.8)
Any complication	388 (9.2)
Any cirrhosis	177 (4.2)
Compensated cirrhosis	177 (4.2)
Decompensated cirrhosis	59 (1.4)
Any malignancy	187 (4.4)
Cholangiocarcinoma	63 (1.5)
Colorectal cancer	50 (1.2)
Gallbladder cancer	28 (0.7)
HCC	17 (0.4)
Pancreatic cancer	35 (0.8)
Liver transplantation	44 (1.0)
Patients who had undergone liver-related procedures, n (%)	
Endoscopic retrograde cholangiopancreatography	312 (7.4)
Cholecystectomy	128 (3.0)
Liver biopsy	97 (2.3)
Endoscopic dilation and stenting	52 (1.2)
Magnetic resonance cholangiopancreatography	49 (1.2)
Patients with comorbidities,^a^ n (%)	
Inflammatory bowel disease	3077 (73.0)
Biliary stone disease	343 (8.1)
Type 2 diabetes	205 (4.9)
Autoimmune hepatitis	152 (3.6)
Type 1 diabetes	92 (2.2)
Medications,^a^^,^^b^ n (%)	
5-aminosalicylic acid and sulfasalazine	1264 (30.0)
Prednisolone	929 (22.1)
Ursodeoxycholic acid	693 (16.4)
Azathioprine	427 (10.1)
Hydroxyzine	228 (5.4)
Budesonide	200 (4.7)
Cholestyramine	105 (2.5)

^a^ Data shown for comorbidities and PSC-related medications are reported in more than 2% of patients.

^b^ Data on filled prescriptions from the Prescribed Drug Register were only available from July 1, 2005.

Abbreviations: HCC, hepatocellular carcinoma; PSC, primary sclerosing cholangitis; SD, standard deviation.

### Healthcare resource use

**FIGURE 2:**
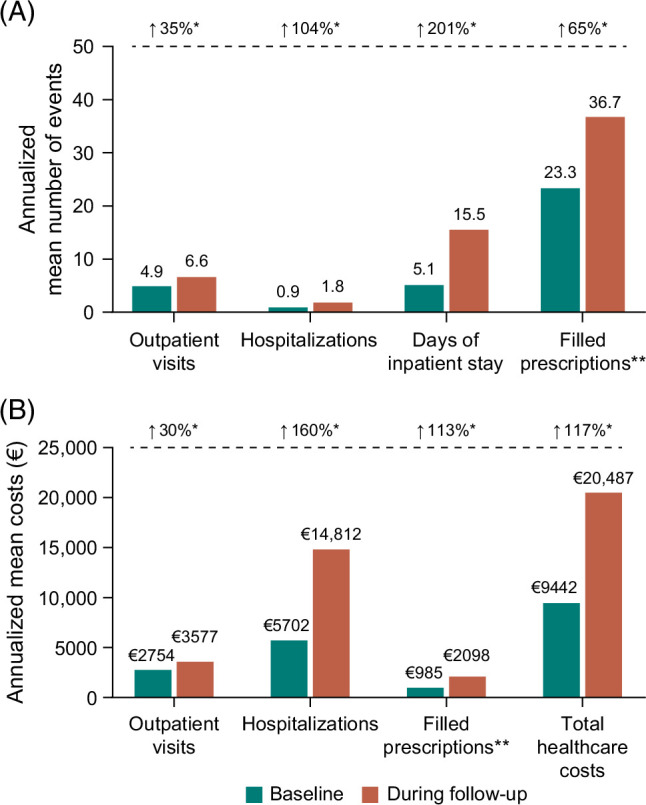
HRU and healthcare costs. Annualized mean HRU (A) and healthcare costs (B) for incident patients with PSC at baseline and follow-up. Median (range) of follow-up was 5.7 (0.1–19.0) y. *Significant increase from baseline to follow-up (*p*<0.0001).**Assessed for the following medications: 5-aminosalicylic acid and sulfasalazine, adalimumab, azathioprine, budesonide, certolizumab, cholestyramine, colestipol, fibrates, golimumab, hydroxyzine, infliximab, mercaptopurine, natalizumab, prednisolone, rifampicin, risankizumab, thioguanine, tofacitinib, ursodeoxycholic acid, ustekinumab, and vedolizumab. Abbreviations: HRU, healthcare resource use; PSC, primary sclerosing cholangitis.

The median (range) duration of follow-up was 5.7 (0.1–19.0) years. The mean (SD) annualized number of outpatient visits significantly increased from 4.9 (7.5) at baseline (the 360 days before the index date) to 6.6 (9.6) during the follow-up period (35% increase, *p*<0.0001) (Figure 2A). Similar increases were observed for the mean (SD) annualized number of hospitalizations [104% increase; 0.9 (1.7) to 1.8 (3.8)], days of inpatient stay [201% increase; 5.1 (12.9) to 15.5 (43.1)], and number of filled prescriptions [65% increase; 22.3 (38.8) to 36.7 (54.3)] (all *p*<0.0001). Median HRU data are provided in Supplemental Table S1, http://links.lww.com/HC9/C196.

Sensitivity analyses indicated that the incidence of “any complication” consistently remained at 1%–3% throughout the follow-up period, and high-cost complications such as liver transplant and cholangiocarcinoma also increased annually by ~0.78% and ~0.56%, respectively (data on file, Gilead Sciences, Inc.).

### Healthcare costs

Mean (SD) annualized total healthcare costs for patients with PSC significantly increased from €9442 (15,678) at baseline to €20,487 (46,893) during the follow-up period, reflecting a 117% increase (*p*<0.0001) (Figure 2B). Mean (SD) annualized costs significantly increased from baseline to during the follow-up period for hospitalizations [160% increase; €5702 (12,706) to €14,812 (44,865)], outpatient visits [30% increase; €2754 (4295) to €3577 (5371)], and filled prescriptions [113% increase; €985 (3882) to €2098 (4167)] (all *p*<0.0001). Hospitalization was the primary contributor to total healthcare costs (60.4% at baseline; 72.3% during follow-up). The median (range) annualized total healthcare costs increased from €4143 (not evaluable–266,955) at baseline to €8118 (69–1,477,216) during the follow-up period (Supplemental Table S1, http://links.lww.com/HC9/C196).

### Risk of complications of PSC and all-cause death

**FIGURE 3:**
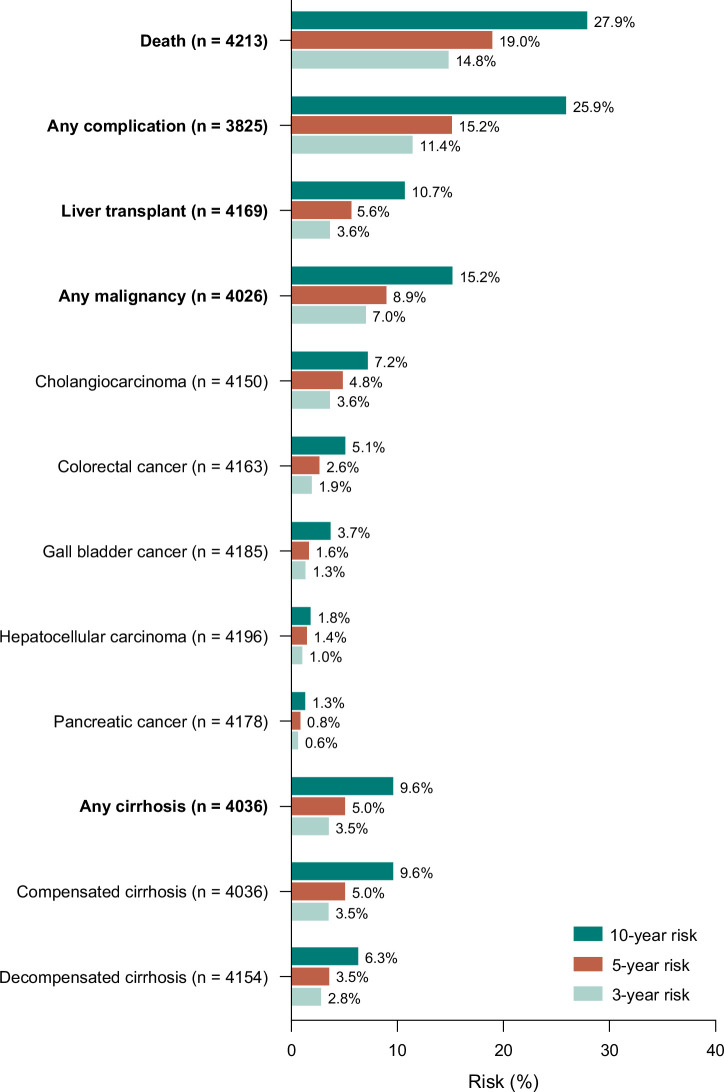
Incidence of complications of PSC or all-cause death. Cumulative incidences for incident patients with PSC during follow-up. Patients have been censored at death, emigration, or the end of the study period. Numbers in brackets show at-risk patients at baseline. Abbreviations: HCC, hepatocellular carcinoma; PSC, primary sclerosing cholangitis.

In total, 675 patients had complications of PSC during follow-up. This included 416 patients who had at least 1 malignancy (cholangiocarcinoma: n=215; colorectal cancer: n=124; gallbladder cancer: n=87; HCC: n=56; pancreatic cancer: n=40), 245 who had cirrhosis (compensated: n=245; decompensated: n=167), and 265 who underwent liver transplantation. The 10-year risk of any complication was 25.9% (95% CI 24.0–27.9) (Figure 3). In total, 935 patients (22.2%) died during follow-up (3306 per 100,000 person-years). The 10-year risk of all-cause death was 27.9% (95% CI 26.2–29.7).

### Terminal care costs

**FIGURE 4:**
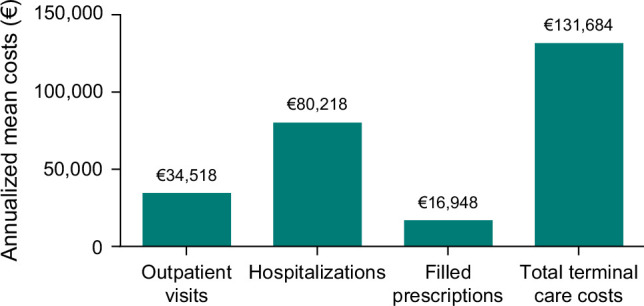
Terminal care costs. Healthcare costs for incident patients with PSC in the 360 days leading up to death. Abbreviation: PSC, primary sclerosing cholangitis.

In the 360 days leading up to death, the mean (SD) total healthcare cost was €131,684 (153,085), which comprised outpatient visits [€34,518 (68,910)], hospitalizations [€80,218 (88,332)], and filled prescriptions [€16,948 (35,164)] (Figure 4).

## DISCUSSION

There is limited real-world evidence evaluating the level of burden and disease progression outcomes in patients with PSC. In this study, registry data were used to provide a comprehensive, updated picture of the clinical characteristics, HRU, healthcare costs, and long-term disease outcomes in patients with PSC in Sweden.

In total, 4213 new diagnoses of PSC between 2002 and 2020 were identified in Sweden, with the prevalence and incidence increasing substantially over this period. In 2020, prevalence and incidence estimates for PSC were 43.9 per 100,000 persons and 3.3 per 100,000 persons/year, respectively. This is in line with a previous study of patients in southern Sweden, which reported an increasing incidence of PSC from 1992 to 2005.^4^ In that study, the prevalence of PSC was 16.2 per 100,000 in the total adult population in 2005.^4^ Similarly, a systematic review of population-based studies reported increases in PSC incidence over time in the USA (1976–2017), the UK (1991–2001), and the Netherlands (2000–2007).^27^ The increase in the number of patients with PSC is probably driven partly by more and better diagnostic testing, as well as increased knowledge and awareness in the physician community, but a true increase in the incidence of PSC cannot be ruled out, as revealed in more recent studies.^27^^–^^30^

The most common medications at baseline in patients with PSC were IBD-specific treatments, consistent with IBD being the most common comorbidity in this cohort, which aligns with what is known in the literature.^2^^,^^8^^,^^31^ Despite being a rare disease, PSC is associated with high HRU and healthcare costs. HRU and healthcare costs associated with PSC in Sweden increased significantly over a median follow-up of 5.7 years. In Sweden, ~11% of the gross domestic product (GDP) was spent on healthcare in 2020, which corresponds to €6120 per patient per year.^32^ The total healthcare costs for patients with PSC more than doubled from baseline to follow-up, to an average of more than €20,000 per patient each year. This was primarily driven by hospitalizations, which accounted for more than 70% of the healthcare costs during the follow-up period. A possible explanation for the large variation in healthcare costs is the small proportion of patients with high healthcare needs, which is evident with a higher mean value (€20,487) over the median value (€8118) during the follow-up period. In a population-based registry study conducted in the Netherlands during 2008–2020, 1208 patients with PSC with a median follow-up of 11.2 years had mean annual medical costs of €12,169.^31^ Although not analyzed in our study, patients who underwent liver transplantation (which falls under hospitalization) during follow-up are likely another driving factor of increasing healthcare costs. In the Netherlands registry study, the annual costs in the year post-liver transplantation amounted to over €126,000 (inclusive of the transplantation), and annual costs after 1 year were over €15,000.^31^

Costs associated with outpatient visits and filled prescriptions also increased significantly from baseline to follow-up. Given that IBD was a comorbid condition in most patients and that the most common medications at baseline were IBD-specific drugs, the increased costs may have been driven by IBD-specific rather than PSC-specific treatment. Indeed, national registry data for patients with IBD in Sweden found that the mean medication costs approximately doubled from 2007 to 2020.^33^ Furthermore, in the Netherlands registry study, costs of IBD medications were similar to the costs of PSC medications.^31^ These findings underscore the need for further research to disentangle PSC-related drivers of HRU and costs from those related to IBD, and highlight an area for further investigation.

PSC is a complex disease with a varied clinical presentation and prognosis.^2^^,^^12^ A recent population-based study in Sweden found a 4-fold higher rate of all-cause death and a 6-fold higher rate of cancer-related death in patients with PSC compared with matched controls from the general population.^34^ Cancer and liver transplantation were high-risk disease outcomes associated with PSC in our study, reported in 15.2% and 10.7% of patients, respectively, at 10 years. The median time to liver transplantation or death was 21 years in a registry study conducted in the Netherlands.^31^ The cumulative 10-year incidence of cholangiocarcinoma in patients with PSC was 7.2%, which was similar to previous reports of 8%–11%.^13^ It is worth noting that liver transplantation is generally contraindicated in patients with cholangiocarcinoma owing to the high risk of recurrence and poor prognosis during immunosuppression after transplantation. Patients also had a high risk of death, which coincided with high terminal care costs. These results highlight the progressive nature of PSC and the unmet need for therapies that can slow disease progression. To address this, continued research into effective treatments and management strategies is warranted.

Strengths of this study include the use of registers with high-quality data and complete coverage of the Swedish population, the large sample size, and the long follow-up period. Retrospectively identifying patients with PSC presented a challenge, owing to potential variability in physician use of ICD-10 codes, particularly at the beginning of the study period when the PSC-specific code (K83.0A) had only recently been introduced. There was significant overlap of patients identified using the PSC-specific ICD-10 code and the combination of the cholangitis and IBD codes, and this was likely representative of the true clinical picture for PSC in Sweden, which also adds to the strength of our study.

There were several study limitations. As with any registry database, the study is subject to data coding limitations and the potential for disease misclassification, leading to underreporting. Although the PSC diagnosis code has not been validated in the Swedish National Patient Register, previous studies have shown high positive predictive values for most disease diagnoses and for cirrhosis-related events.^35^^,^^36^ Patients with autoimmune hepatitis, other liver diseases, and alcohol use were not excluded, which may risk misclassification of other diseases as PSC. The lack of primary care and indirect costs (eg, work loss or reduced quality of life) would have precluded a true reflection of the costs associated with PSC; however, these costs would have potentially captured the costs for managing other comorbidities, especially given the high prevalence of IBD in our cohort. Notably, there was a small number of patients who underwent liver transplantation in our incident cohort that may have impacted the increased healthcare costs, owing to different HRU and associated costs compared with patients who had not yet had a liver transplantation. In addition, although imaging (eg, by magnetic resonance imaging, magnetic resonance cholangiopancreatography, or ultrasound) is an important component of PSC care, costs for these procedures could not be individually determined from the data available. However, these costs are captured indirectly through the DRG codes used for cost estimation and are therefore still reflected in the overall cost estimates. Another limitation is that the cumulative incidence of the first complication event did not account for the competing risk of other complications, which can lead to an overestimation of the incidence of complications. In addition, there is a risk that right censoring impacted costs in this study. However, the sensitivity analysis showed a consistent incidence of any complication and a stable annual increase in incidence of high-cost complications, reducing the likelihood that right censoring caused overestimation or underestimation of mean annual costs or HRU.

## CONCLUSIONS

Overall, the real-world data on the disease and economic burden of PSC reported in this study may provide insights for resource allocation and funding by healthcare policymakers and clinicians at a national level. Although PSC is considered a rare disease, this study showed that the prevalence and incidence of PSC have increased significantly in Sweden. HRU and healthcare costs associated with PSC were substantial. Early identification and effective therapies are needed to reduce the risk of disease progression, improve prognosis, and mitigate the clinical and economic burden of PSC.

## Supplementary Material

## References

[1] Rabiee A Silveira MG . Primary sclerosing cholangitis. Transl Gastroenterol Hepatol. 2021;6:29. 33824933 10.21037/tgh-20-266 PMC7829069

[2] Manns MP Bergquist A Karlsen TH Levy C Muir AJ Ponsioen C . Primary sclerosing cholangitis. Nat Rev Dis Primers. 2025;11:18. 40082445 10.1038/s41572-025-00600-x

[3] Boonstra K Beuers U Ponsioen CY . Epidemiology of primary sclerosing cholangitis and primary biliary cirrhosis: A systematic review. J Hepatol. 2012;56:1181–1188. 22245904 10.1016/j.jhep.2011.10.025

[4] Lindkvist B Benito de Valle M Gullberg B Björnsson E . Incidence and prevalence of primary sclerosing cholangitis in a defined adult population in Sweden. Hepatology. 2010;52:571–577. 20683956 10.1002/hep.23678

[5] Karlsen TH Folseraas T Thorburn D Vesterhus M . Primary sclerosing cholangitis - A comprehensive review. J Hepatol. 2017;67:1298–1323. 28802875 10.1016/j.jhep.2017.07.022

[6] Dyson JK Beuers U Jones DEJ Lohse AW Hudson M . Primary sclerosing cholangitis. Lancet. 2018;391:2547–2559. 29452711 10.1016/S0140-6736(18)30300-3

[7] Lamberts LE Janse M Haagsma EB van den Berg AP Weersma RK . Immune-mediated diseases in primary sclerosing cholangitis. Dig Liver Dis. 2011;43:802–806. 21700515 10.1016/j.dld.2011.05.009

[8] Mertz A Nguyen NA Katsanos KH Kwok RM . Primary sclerosing cholangitis and inflammatory bowel disease comorbidity: An update of the evidence. Ann Gastroenterol. 2019;32:124–133. 30837784 10.20524/aog.2019.0344 PMC6394256

[9] Cançado GGL Hirschfield GM . Management of primary sclerosing cholangitis: Current state-of-the-art. Hepatol Commun. 2024;8:e0590. 39774274 10.1097/HC9.0000000000000590 PMC11567710

[10] Lundberg Bave A Bergquist A Bottai M Warnqvist A von Seth E Nordenvall C . Increased risk of cancer in patients with primary sclerosing cholangitis. Hepatol Int. 2021;15:1174–1182. 34357546 10.1007/s12072-021-10214-6 PMC8514354

[12] Chapman MH Thorburn D Hirschfield GM Webster GGJ Rushbrook SM Alexander G . British Society of Gastroenterology and UK-PSC guidelines for the diagnosis and management of primary sclerosing cholangitis. Gut. 2019;68:1356–1378. 31154395 10.1136/gutjnl-2018-317993 PMC6691863

[11] Weismüller TJ Trivedi PJ Bergquist A Imam M Lenzen H Ponsioen CY . Patient age, sex, and inflammatory bowel disease phenotype associate with course of primary sclerosing cholangitis. Gastroenterology. 2017;152:1975–1984.e8. 28274849 10.1053/j.gastro.2017.02.038 PMC5546611

[13] Song J Li Y Bowlus CL Yang G Leung PSC Gershwin ME . Cholangiocarcinoma in patients with primary sclerosing cholangitis (PSC): A comprehensive review. Clin Rev Allergy Immunol. 2020;58:134–149. 31463807 10.1007/s12016-019-08764-7

[14] Villard C Friis-Liby I Rorsman F Said K Warnqvist A Cornillet M . Prospective surveillance for cholangiocarcinoma in unselected individuals with primary sclerosing cholangitis. J Hepatol. 2023;78:604–613. 36410555 10.1016/j.jhep.2022.11.011

[15] Vesterhus M Karlsen TH . Emerging therapies in primary sclerosing cholangitis: Pathophysiological basis and clinical opportunities. J Gastroenterol. 2020;55:588–614. 32222826 10.1007/s00535-020-01681-z PMC7242240

[16] Bowlus CL Arrivé L Bergquist A Deneau M Forman L Ilyas SI . AASLD practice guidance on primary sclerosing cholangitis and cholangiocarcinoma. Hepatology. 2023;77:659–702. 36083140 10.1002/hep.32771

[17] Chazouilleres O Beuers U Bergquist A Karlsen TH Levy C Samyn M . European Association for the Study of the Liver . EASL Clinical Practice Guidelines on sclerosing cholangitis. J Hepatol. 2022;77:761–806. 35738507 10.1016/j.jhep.2022.05.011

[18] Chazouilleres O Beuers U Bergquist A Karlsen TH Levy C Samyn M . European Association for the Study of the Liver . Corrigendum to “EASL clinical practice guidelines on sclerosing cholangitis” [J Hepatol (77) 761–806]. J Hepatol. 2023;79:1339. 37735011 10.1016/j.jhep.2023.09.005

[19] Abbas N Quraishi MN Trivedi P . Emerging drugs for the treatment of primary sclerosing cholangitis. Curr Opin Pharmacol. 2022;62:23–35. 34894541 10.1016/j.coph.2021.11.003

[20] Steenstraten IC Sebib Korkmaz K Trivedi PJ Inderson A van Hoek B Rodriguez Girondo MDM . Systematic review with meta-analysis: Risk factors for recurrent primary sclerosing cholangitis after liver transplantation. Aliment Pharmacol Ther. 2019;49:636–643. 30740723 10.1111/apt.15148 PMC6593422

[21] Molodecky NA Kareemi H Parab R Barkema HW Quan H Myers RP . Incidence of primary sclerosing cholangitis: A systematic review and meta-analysis. Hepatology. 2011;53:1590–1599. 21351115 10.1002/hep.24247

[22] Stokkeland K Höijer J Bottai M Söderberg-Löfdal K Bergquist A . Statin use is associated with improved outcomes of patients with primary sclerosing cholangitis. Clin Gastroenterol Hepatol. 2019;17:1860–1866.e1. 30448601 10.1016/j.cgh.2018.11.002

[23] Quan H Li B Couris CM Fushimi K Graham P Hider P . Updating and validating the Charlson Comorbidity Index and score for risk adjustment in hospital discharge abstracts using data from 6 countries. Am J Epidemiol. 2011;173:676–682. 21330339 10.1093/aje/kwq433

[24] Socialstyrelsen . Diagnosis-related groups (Swedish). Accessed August 5, 2025. https://www.socialstyrelsen.se/statistik-och-data/klassifikationer-och-koder/drg/

[25] European Central Bank . Swedish krona (SEK). Accessed July 24, 2025. https://www.ecb.europa.eu/stats/policy_and_exchange_rates/euro_reference_exchange_rates/html/eurofxref-graph-sek.en.html

[26] Riksbank . Interest rates and exchange rates. Accessed July 24, 2025. https://www.riksbank.se/en-gb/statistics/interest-rates-and-exchange-rates/search-interest-rates-and-exchange-rates/

[27] Trivedi PJ Bowlus CL Yimam KK Razavi H Estes C . Epidemiology, natural history, and outcomes of primary sclerosing cholangitis: A systematic review of population-based studies. Clin Gastroenterol Hepatol. 2022;20:1687–1700. e4. 34474162 10.1016/j.cgh.2021.08.039

[28] Carbone M Kodra Y Rocchetti A Manno V Minelli G Gerussi A . Primary sclerosing cholangitis: Burden of disease and mortality using data from the national rare diseases registry in Italy. Int J Environ Res Public Health. 2020;17:3095. 32365682 10.3390/ijerph17093095 PMC7246900

[29] Cooper J Markovinovic A Coward S Herauf M Shaheen AA Swain M . Incidence and prevalence of primary sclerosing cholangitis: A meta-analysis of population-based studies. Inflamm Bowel Dis. 2024;30:2019–2026. 38052097 10.1093/ibd/izad276 PMC11532590

[30] Liang H Manne S Shick J Lissoos T Dolin P . Incidence, prevalence, and natural history of primary sclerosing cholangitis in the United Kingdom. Medicine (Baltimore). 2017;96:e7116. 28614231 10.1097/MD.0000000000007116 PMC5478316

[31] van Munster KN Mol B Goet JC van Munster SN Weersma RK de Vries AC . Disease burden in primary sclerosing cholangitis in the Netherlands: A long-term follow-up study. Liver Int. 2023;43:639–648. 36328957 10.1111/liv.15471

[32] Statistics Sweden . 2025. Accessed March 11, 2025. https://www.scb.se/

[33] Everhov ÅH Söderling J Befrits G Khalili H Bröms G Neovius M . Increasing healthcare costs in inflammatory bowel disease 2007–2020 in Sweden. Aliment Pharmacol Ther. 2023;58:692–703. 37594381 10.1111/apt.17675

[34] Lasyte I Widman L Bergquist A Hagström H . Mortality in autoimmune liver disease in Sweden: A population-based cohort study of 9,654 patients. Liver Int. 2025;45:e70007. 39840802 10.1111/liv.70007 PMC11752691

[35] Bengtsson B Askling J Ludvigsson JF Hagström H . Validity of administrative codes associated with cirrhosis in Sweden. Scand J Gastroenterol. 2020;55:1205–1210. 32960654 10.1080/00365521.2020.1820566

[36] Ludvigsson JF Andersson E Ekbom A Feychting M Kim JL Reuterwall C . External review and validation of the Swedish National Inpatient Register. BMC Public Health. 2011;11:450. 21658213 10.1186/1471-2458-11-450 PMC3142234

