# Supplementary material for: Burden of primary sclerosing cholangitis in Sweden (2002–2020): Incidence, outcomes, healthcare utilization, and costs
Source: Hepatol Commun. 2025 Dec 12;10(1):e0858. doi: 10.1097/HC9.0000000000000858 (PMC12705051; doi:10.1097/HC9.0000000000000858)
Supplement: Supplementary file 1 [file hc9-10-e0858-s001.docx]

**Burden of primary sclerosing cholangitis in Sweden (2002–2020): incidence, outcomes, healthcare utilization, and costs**

Annika Bergquist^1,2^ | Nandita Kachru^3^ | Martina Aldvén^4^ | Oskar Ström^4^ | Helena Skröder^4^ | Emilie Toresson Grip^1,4^ | Hannes Hagström^1,2^

^1^Department of Medicine, Huddinge, Karolinska Institutet, Stockholm, Sweden
^2^Division of Hepatology, Department of Upper GI Diseases, Karolinska University Hospital, Stockholm, Sweden
^3^Gilead Sciences, Inc., Foster City, CA, USA
^4^Quantify Research AB, Stockholm, Sweden

# Supplementary

**Supplementary Table 1** Annualized median HRU and healthcare costs for incident patients at baseline and follow-up. Abbreviations: EUR, euro; HRU, healthcare resource use; NE, not evaluable; USD, United States Dollar.

|  | **Baseline period** | **Follow-up period** |
| --- | --- | --- |
| **HRU, median (range)** | | |
| Number of outpatient visits | 3.0 (0–204) | 4.4 (0–205) |
| Number of hospitalizations | 0 (0–31) | 0.5 (0–49) |
| Days of inpatient stay | 0 (0–237) | 2.1 (0–365) |
| Number of filled prescriptions | 11 (0–594) | 20.3 (0–548) |
| **Healthcare costs (EUR), median (range)** | | |
| Outpatient visits | €1,805 (NE–107,516) | €2,298 (NE–101,916) |
| Hospitalizations | NE (NE–206,391) | €3,384 (NE–1,477,216) |
| Fill prescriptions | €288 (NE–131,198) | €984 (NE–113,055) |
| Total costs | €4,143 (NE–266,955) | €8,118 (69–1,477,216) |
| **Healthcare costs (USD), median (range)** | | |
| Outpatient visits | $2,134 (NE–127,172) | $2,717 (NE–120,496) |
| Hospitalizations | NE (NE–240,499) | $3,998 (NE–1,748,140) |
| Fill prescriptions | $340 (NE–154,983) | $1,164 (NE–131,954) |
| Total costs | $ 4,903 (NE–315,163) | $ 9,615 (82–1,748,140) |
